# Study of transport, tissue distribution, depletion, and hepatotoxicity of Cyadox, a quinoxaline-1,4-dioxide derivative

**DOI:** 10.3389/fphar.2024.1401275

**Published:** 2024-09-23

**Authors:** Zhu Tao, Changchun Li, Aiqun Zhang, Zhilin Zhang, Jing Huang, Sechenchogt Harnud

**Affiliations:** ^1^ Research Center for Ecotoxicology and Food Safety, Hubei Engineering University, Xiaogan, China; ^2^ College of Life Science and Technology, Hubei Engineering University, Xiaogan, China; ^3^ Hubei Province Research Center of Engineering Technology for Utilization of Botanical Functional Ingredients, Hubei Engineering University, Xiaogan, China; ^4^ Hubei Key Laboratory of Quality Control of Characteristic Fruits and Vegetables, Hubei Engineering University, Xiaogan, China

**Keywords:** cyadox, distribution, docking, radioisotopic tracing, hepatotoxicity

## Abstract

**Background:**

Cyadox (CYA) is a derivative of quinoxaline 1,4-dioxide and a safe and effective synthetic antibacterial agent.

**Objective:**

This study aimed to explore the drug transport in blood, distribution, depletion and hepatotoxicity of drugs in animals.

**Methods:**

The transport of CYA in blood was studied using fluorescence, circular dichroism (CD) and molecular docking methods. Tissue distribution and depletion of CYA in rats were evaluated following oral administration of [3H]-CYA at different doses. Hepatotoxicity of drugs evaluated by transcriptomics.

**Results:**

During transport in the bloodstream, the drug binds to bovine serum albumin (BSA) by hydrogen bonding and has only one binding site. Hydrogen bonds were formed between O (2) of CYA and ARG208, O (3) of CYA and LEU480, VAL481. The secondary protein conformation of BSA changed after binding with an increase in α-helix and a decrease in β-strand. After a single oral administration of [^3^H]-CYA, it was excreted rapidly within 7 days, with 34.81% from the urine and 60.25% from the feces. Higher and sustained levels of radioactivity were detected in the liver during the post-dose period, suggesting that the drug may concentrate in the liver. The transcriptomic data indicates that CYA exhibits low hepatotoxicity. However, there are indications that it may have an impact on steroid biosynthesis.

**Conclusion:**

This study could serve as a basis for conducting further studies on the use of CYA in food animals and improving the pharmacologic, pharmacokinetic, and toxicologic effects of CYA on food animals.

## 1 Introduction

Quinoxaline 1,4-dioxide derivatives (QdNOs) have been widely used as antimicrobial growth-promoting agents or effective therapeutic drugs in animals for decades. Olaquindox and carbadox are common QdNOs banned or limited in animal husbandry in many countries owing to their potential toxicity (Wang et al., 2015; Ihsan et al., 2010; Ihsan et al., 2013; Huang et al., 2008). Cyadox ((2-formylquinoxaline)-N1,N4-dioxide cyanoacetylhydrazone; CYA) is a QdNO that is considered safe and effective as a synthetic antibacterial agent. It can promote animal growth and improve feed efficiency with a potential to be used in livestock in the future (Huang et al., 2016; Liu et al., 2017; Laulloo et al., 2022). For a veterinary drug to be registered in China, it must undergo extensive pharmacological and toxicological studies to understand its tissue distribution, depletion, and excretion and assess its safety in food animals.

Serum albumin is the most abundant protein in plasma with several physiological and pharmacological functions. Most drugs travel in the plasma and reach the target tissues by binding to serum albumin. The interaction between CYA and plasma albumin must be analyzed to obtain further information about the distribution of CYA. Up to now, several spectrophotometric methods, including fluorescence, UV-Vis, circular dichroism (CD), and Fourier-transform infrared spectroscopy (FT-IR), have been used to study the interaction of drugs with proteins to clarify the conformational change of proteins (Liu et al., 2009; Zheng et al., 2011; Xu et al., 2011; Wu et al., 2012). Molecular docking is also used to explore the binding between drugs and serum albumin (Li et al., 2013). After binding to a drug, the spectrum of the plasma protein changes in most cases (Huang et al., 2018). Fluorescence and CD spectroscopy have been widely utilized to evaluate binding mode and constants owing to their exceptional sensitivity, selectivity, convenience, and abundant theoretical foundation (Marathe et al., 2004).

Radiolabeled compounds are excellent tools for conducting drug disposition studies during drug discovery and development. The radioisotope tracer technique offers a unique mode of obtaining quantitative information of total drug-related molecules and their decomposition products, metabolites, and other substances (Ashraf et al., 2022). Several recent studies have described the pharmacokinetics and tissue depletion of CYA and its metabolites in various animals following oral administration (Laulloo et al., 2022; Huang et al., 2022; Lu et al., 2022). However, the effects of long-term drug presence at tissue sites have not been investigated. Therefore, toxicity studies of drugs in tissues are justified.

In the present study, the interaction between bovine serum albumin (BSA) with CYA was primarily studied using fluorescence, CD spectroscopy, and molecular docking. The present study investigated the tissue distribution, depletion, and hepatotoxicity of CYA *in vivo*. The results would describe the comprehensive disposition of CYA, which could provide abundant reference data for the study of CYA *in vivo* and *in vitro* and improve the explanation for the toxicities and food safety evaluation of CYA in food animals.

## 2 Materials and methods

### 2.1 Chemicals

**FIGURE 1:**
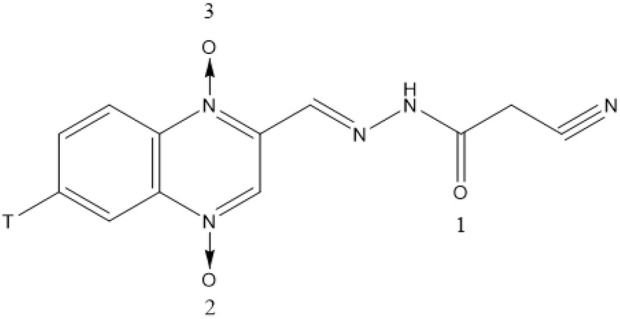
Structure of [^3^H]-CYA (T: Tritium).

CYA (purity ≥ 99%) and [^3^H]-CYA (Figure 1) were synthesized by the Institute of Veterinary Pharmaceuticals of Huazhong Agricultural University (Wuhan, People’s Republic of China) (Jia et al., 2024). [^3^H]-CYA had a specific activity of 2.08 Ci/mmol and ≥ 99% radiochemically purity on HPLC-SLC and preserved at specific activity of 39.71 mCi/mmol after diluted with unlabeled CYA. Exchange of the ^3^H label with water was confirmed. Solvable^TM^ (tissue-solubilizing fluid), Ultima Gold, Monophase-S (Liquid scintillation cocktails) and Combustaid were purchased from PerkinElmer Life and Analytical Sciences (Groningen, Netherlands; Waltham, MA, United States). All chemicals were of reagent grade.

### 2.2 Fluorescence spectroscopy

The Albumin Bovine V from BSA (Huashun Company, Wuhan, China) solution (1.0 × 10^−6^ M based on its molecular weight of 68,000) was prepared in a PBS buffer with pH 7.4. The concentration of CYA was varied at 0, 0.33, 0.66, 1.0, 1.33, 1.66, and 2.0 × 10^−6^ M.

Steady-state fluorescence spectra were performed on a FP-6500 spectrofluorimeter (Jasco, Japan) equipped with a thermostatic bath and 1.0 cm quartz cell. Fluorescence emission spectra were recorded at 300, 305, and 310 K in the range of 295 nm–500 nm. The excitation and emission slits with a band pass of 5 nm were used for all the measurements.

Synchronous fluorescence spectra were performed when the D-values between the excitation and emission wavelengths were set at 15 and 60 nm. Fluorescence excitation spectra were recorded at 302, 307, and 312 K in the range of 200–400 nm.

### 2.3 Circular dichroism spectroscopy

For CD studies, the final concentration of BSA and CYA was 1.0 × 10^−6^ M. The CD spectra were recorded at 312 K with a Jasco-810 spectropolarimeter (Jasco, Japan) using a 0.1 cm quartz cuvette. Three scans were accumulated at a scan speed of 200 nm min^−1^, with data being collected every 0.2 nm from 200 nm to 260 nm at 312 K.

### 2.4 Molecular docking

Docking calculations were performed via AutoDock 4.2. PDBQT files containing the charge and atom-type information of CYA and BSA (PDB ID:4F5S) were prepared by AutoDock Tools. Genetic algorithm (GA) was used to optimize the ligand conformation.

### 2.5 Animals

Thirty 7-week-old male and female Wistar rats weighing 202 ± 18 g and 4-week-old male and female KM mice weighing 20 ± 2 g were purchased from Hubei Center for Disease Prevention and Control (Wuhan, People’s Republic of China). After a 7-day acclimatization period, the animals were housed individually in stainless steel metabolism cages. The animals were kept in an environment maintained at a temperature of 20°C–26°C, relative humidity of 40%–70%, and a 12 h light/dark cycle. Feed and water were provided *ad libitum*. All animals were monitored through the day, and individual body weights were recorded prior to the study. All studies were approved by the experimental animal ethics committee of Hubei Engineering University.

### 2.6 Drug distribution, depletion and excretion

For the tissue distribution study, 24 rats were divided into six groups, one group as a blank control and five groups as five time point groups. Each time point group consisted of 4 rats (2 male and 2 female) administered [^3^H]-CYA orally by gavage at 10 mg/kg bw (corresponded to a feed inclusion of approximately 100 mg/kg dose) for seven consecutive days, respectively. Dosing suspensions of [^3^H]-CYA (2.08 Ci/mmol) were prepared in 0.5% aqueous carboxymethyl cellulose (CMC) at 4 mg/mL after being diluted with non-radiolabeled CYA to a specific activity of 9,044 dpm/µg. Another group of 4 rats (2 male and 2 female) was fed separately from the radioactive administration group for the blank control under the same conditions, sacrificed at 0 day. One group of 2 male and 2 female rats was randomly selected to be slaughtered at each time point (0.25, 1, 3, 7, and 14 days) after withdrawal. The rats were dissected, and blood, muscle, heart, liver, lung, spleen, kidney, skin, stomach, large intestine, small intestine, brain, cecum, testicle (or ovary), bladder, and bone (thigh bone) were collected separately and stored at −20°C until analysis. The weight of each tissue was recorded. Gastrointestinal tracts were stripped of their contents.

For the excretion study, a group of six rats (3 male and 3 female) were housed individually in metabolism cages, which allowed separate collection of urine and feces, and then administered with a single dose of 10 mg/kg bw (corresponded to a feed inclusion of approximately 100 mg/kg dose) [^3^H]-CYA by oral gavage. Dosing suspensions of [^3^H]-CYA were prepared in 0.5% aqueous CMC at 2 mg/mL after [^3^H]-CYA (2.08 Ci/mmol) was diluted with non-radiolabeled CYA to a specific activity of 4.52 × 10^4^ dpm/µg. Urine and feces were collected from intact animals for 7 days at 0–12, 12–24, 24–48, 48–72, 72–96, 96–120, 120–144, and 144–168 h after the dose, and the total weight of the urine and feces was recorded after each collection. At the end of the study, the animals are anesthetized, and once it's confirmed that they have lost consciousness, they are euthanized by exsanguination. The samples were stored at −20°C until analysis. Contents of gastrointestinal tracts were pooled with feces of 144–168 h time point. Each metabolism cage was thoroughly rinsed with a mixture of methanol/water (1:1; V/V), and the rinse solvent was collected and weighed. Each cage rinse was stirred thoroughly, 500 mL of subsamples were removed, and radioactivities were analyzed immediately. One animal per gender was administered with non-radiolabeled CYA at the same dose, and animals were sacrificed at 6 h post-dose. Urine, feces, and tissues were collected for the blank controls.

### 2.7 Determination of total radioactivity

For radioactivity detection, the samples were combusted by PerkinElmer Oxidizer 307 (PerkinElmer Life and Analytical Sciences). ^3^H_2_O was collected into 20 mL of scintillant (PerkinElmer Life and Analytical Sciences) with 10 mL of Monophase S, and sample radioassays were quantitated by PerkinElmer^®^ TriCarb^®^ 2900 (PerkinElmer Life and Analytical Sciences) with low-level quench-correction. Counting efficiency was above 65%.

Tissue samples for each sampling time from all animals were homogenized, aliquots of blood and urine were mixed thoroughly, and feces were homogenized in methanol/water (50:50; V/V). Samples of the blood (500 μL), urine (500 μL) feces (500 mg), bone, and all tissues (100–500 mg) were combusted in PerkinElmer Oxidizer 307 after being transferred into tared cones and pads (PerkinElmer Life and Analytical Sciences) and added an aliquot of Combustaid (200 μL; PerkinElmer Life and Analytical Sciences). Radioactivity in the combustion products was determined by collecting ^3^H_2_O into distilled water followed by liquid scintillation counting. Combustion efficiency was determined daily, prior to the combustion of study samples, by using a diluted dose formulation or ^3^H standard. The measured radioactivity content in the combusted samples was adjusted using the combustion efficiency values. Samples were analyzed for radioactivity for 5 min. Total residue concentrations were calculated from the specific activity and radioactivity per mass or volume and expressed as molar equivalents of CYA.

### 2.8 Studies on hepatotoxicity by transcriptomics

For the hepatotoxicity study, a group of four mice (2 male and 2 female, KM) were administered CYA at a dose of 1,000 mg/kg bw. Another group of four mice received saline as a control. After 7 days, the mice were anaesthetised, sacrificed and their livers collected. A total of eight samples from 2 groups of the obtained livers were sent to Majorbio Technology Co., Ltd. (Shanghai, China) for testing, divided into the blank group (control; A1, A2, A3, A4) and the drug group administered CYA (drug; B1, B2, B3, B4), with 4 samples in each group. Sequencing was performed using the Meiji Medical Reference Transcriptome Sequencing Platform to complete the transcriptome analysis of eight samples. The raw reads were deposited into the NCBI Sequence Read Archive (SRA) database (BioProject: Number: PRJNA1082257).

## 3 Results

### 3.1 Fluorescence spectroscopy and binding property of CYA to BSA

**FIGURE 2:**
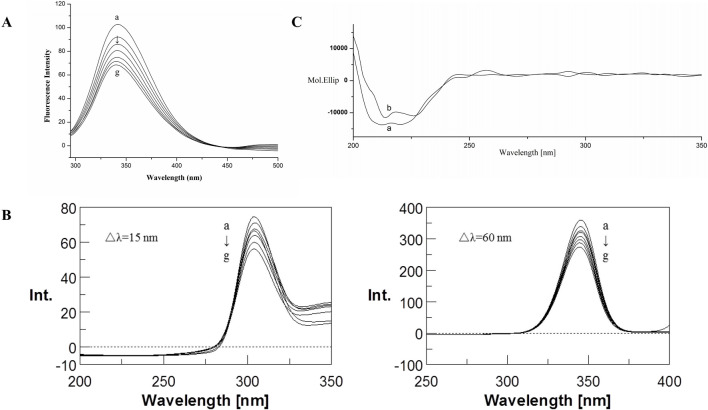
Fluorescence emission spectra **(A)** and Synchronous fluorescence spectra **(B)** of the BSA–CYA system. The concentrations of CYA and BSA for fluorescence spectra was varied at 0, 1, 2, 3, 4, 5, and 6 × 10^−6^ M (a–g, respectively), c (BSA) = 1.0 × 10^−6^ M, pH 7.4, T = 312 (K) **(C)** CD spectra of the BSA–CYA system. The concentrations of CYA and BSA for CD spectra were as follows: **(A)** c (BSA) = 1.0 × 10^−6^ M; **(B)** 1.0 × 10^−6^ M BSA in the presence of 1.0 × 10^−6^ M CYA; pH 7.4, T = 312 (K).

The fluorescence emission spectra of BSA in the absence and presence of CYA in the PBS buffer with pH 7.4 are shown in Figure 2. The BSA protein showed a strong emission peak at 341 nm on excitation wavelength at 280 nm. Thus, the BSA fluorescence intensity decreased in the presence of CYA, indicating that CYA interacted with BSA.

The Stern–Volmer equation below was used to analyze the fluorescence quenching data and confirm the quenching mechanism (Zhou et al., 2022): $\frac{F_{0}}{F}=1+K_{SV}\left[Q\right]=1+k_{q}\tau_{0}\left[Q\right]$ , where *F*_0_ and *F* are the fluorescence intensities before and after the addition of the quencher CYA, *K*_*sv*_ is the Stern–Volmer quenching constant, *k*_q_ is the bimolecular quenching constant, *τ*_0_ is the lifetime of the fluorophore in the absence of the quencher (10^−8^ s), and [Q] is the concentration of the quencher. The calculated *K*_sv_ and *k*_q_ values are summarized in Table 1. The Stern–Volmer quenching constant *K*_sv_ is inversely correlated with temperature, and the *k*_q_ values are much greater than the maximum scattering collisional quenching constant (2.0 × 10^10^ M^−1^S^−1^). Our data suggest that the fluorescence quenching of BSA is initiated by BSA–CYA complex formation rather than dynamic collision.

**TABLE 1:** Stern-Volmer quenching constants of the BSA-CYA system at different temperatures.

T (K)	K_sv_ (M^−1^)	k_q_ (M^−1^S^−1^)	R^2^
302	8.65 × 10^4^	8.65 × 10^12^	0.9978
307	4.07 × 10^4^	4.07 × 10^12^	0.9987
312	3.36 × 10^4^	3.36 × 10^12^	0.9966

For static quenching, the equilibrium between the free and bound molecules is given by the following equation: $\log\frac{F_{0}-F}{F}=\log K_{A}+n\log\left[Q\right]$ , where *K*_*A*_ is the binding constant, and *n* is the number of binding sites. The results are presented in Table 2. The *K*_*A*_ values decreased with increasing temperature, indicating that the BSA–CYA complex was unstable and partly decomposed when the temperature was increased. Moreover, the values of *n* were approximately equal to 1, which indicated the presence of one binding site in the BSA–CYA system.

**TABLE 2:** Binding constants K_A_ and binding sites n of the BSA-CYA system at different temperatures.

T (K)	K_A_ (L·mol^−1^)	n	R^2^
302	1.75 × 10^5^	1.139	0.9926
307	0.97 × 10^4^	0.915	0.9926
312	5.62 × 10^3^	0.931	0.9982

### 3.2 Synchronous fluorescence

Synchronous fluorescence can provide the characteristic information of tyrosine residues in BSA when the D-values between excitation and emission wavelengths were set at 15 nm, the same as tryptophan residues when the D-values were set at 60 nm. If an obvious shift was observed at maximum emission, then the polarity around the protein residues changed.

The influence of CYA on the synchronous fluorescence spectra of BSA is shown in Figure 2B. An obvious red shift of tryptophan residues occurred, suggesting that the polarity around the tryptophan residues changed. The tryptophan residues participated in the interaction between CYA and BSA. By contrast, the maximum emission wavelength of tyrosine residues showed no significant shift, indicating that the polarity of the tyrosine residues did not change during the binding process.

### 3.3 Circular dichroism spectra

The CD spectra of BSA in the absence and presence of CYA are shown in Figure 2C, which indicated that the binding of CYA to BSA induced a significant conformational change. The secondary protein conformation of BSA was found to be 20% α-helix, 35% β-strand, 19% turns, and 26% unordered. When was CYA added into the system, the secondary structure of BSA changed into be 21% α-helix, 28% β-strand, 26% turns, and 25% unordered. The secondary protein conformation of BSA showed an α-helix increase and a β-strand decrease after CYA binding to BSA.

### 3.4 Molecular docking results

**FIGURE 3:**
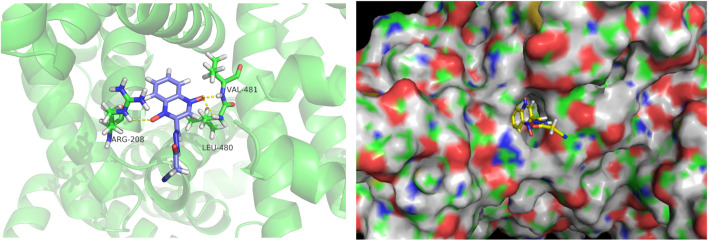
Molecular docking diagrams depicting the binding of BSA with CYA at the lowest binding energy, generated using AutoDock. The image was visualized using PyMol. The left figure displays the binding information of CYA and BSA in cartoon mode, showing the hydrogen bonds formed between O (2) of CYA and ARG208, O (3) of CYA and LEU480, VAL481. The right figure is presented in surface mode, clearly illustrating CYA docked within the binding pocket of BSA.

Docking calculations were performed using AutoDock. The lowest binding free energy of CYA was −5.77 kcal mol^−1^. The docking results are shown in Figure 3. The hydrogen bonds formed between O (2) of CYA and ARG208, O (3) of CYA and LEU480, VAL481. The formation of new hydrogen bonds increased the stability of the CYA–BSA complex. The 3D crystal structure of BSA showed the presence of a pocket-like structure in which the drug can be embedded.

### 3.5 Excretion of CYA

**FIGURE 4:**
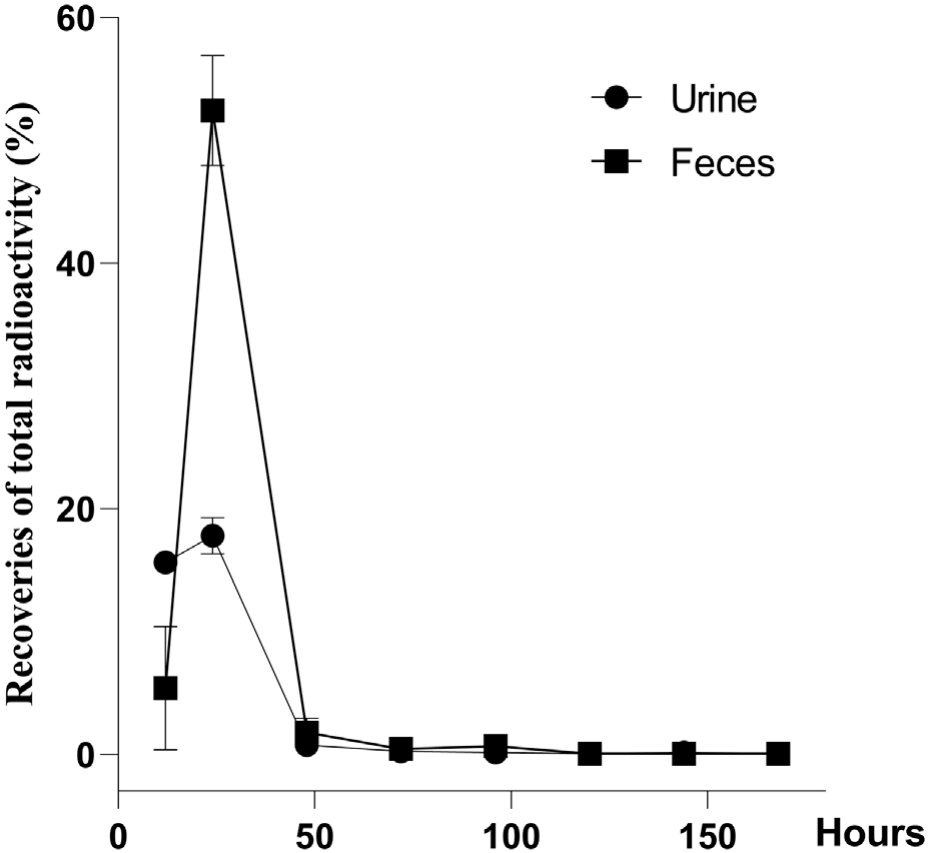
Recoveries of total radioactivity after a single oral administration of [3H]-CYA at 10 mg/kg bw (n = 6). Urine and feces were collected from intact animals for 7 days at 0–12, 12–24, 24–48, 48–72, 72–96, 96–120, 120–144, and 144–168 h after the dose, and the total weight of the urine and feces was recorded after each collection. The radioactivity was mainly excreted in the feces (60.25%) and then in the urine (34.81%).

The recovery of total radioactivity in urine, feces, carcass, and cage rinse after a single oral administration of [^3^H]-CYA in rats is summarized in Figure 4. Overall recoveries showed an average of 96.22% within 7 days (168 h) of withdrawal. The radioactivity was mainly excreted in the feces (60.25%) and then in the urine (34.81%). The radioactivity remaining in the carcass was 0.38%, and the radioactivity of the cage rinse was 0.78%. In addition, 91.28% of the administration amount of the drug-related radioactivity was excreted during the first 24 h after administration, and that excreted at 0–12 and 12–24 h post-dose accounted for 21.03% and 70.25% of the total dose, respectively.

### 3.6 Tissue distribution and depletion

Tissue distribution and depletion of CYA in the rats orally administered with [^3^H]-CYA for seven consecutive days at 10 mg/kg bw are presented in Table 3. Drug-related radioactivity was distributed rapidly in any of the analyzed organs or tissues, with maximum concentrations achieved at 6 h in all detected organs or tissues. At 14 days after dose administration, radioactivity was undetectable in any of the analyzed blood, organs, or tissues, indicating that the drug was eliminated rapidly in the rats. Radioactivity in all of the analyzed organs and tissues declined rapidly after 6 h withdrawal. In addition, the total radioactivity in the alimentary canal tissues, such as cecum, large intestine, small intestine, and stomach, were relatively high at earlier time-points, whereas residual time was short. From 1 to 7 days, the radioactivity in the liver is highest, indicating that the drug may be present in the liver for a long time.

**TABLE 3:** Distribution, depletion and elimination half-life (t_1/2_) of CYA of rats after an oral administration of [^3^H]-CYA for seven consecutive days at 10 mg/kg·bw (The unit for [^3^H]-CYA content in tissues is µg/kg; n = 4).

Tissues	Tissue distribution and depletion of CYA (means ± SD, µg/kg in tissues)					t_1/2_ (h)
	6 h	1 d	3 d	7 d	14 d	
Blood	1552.98 ± 133.22	785.75 ± 56.13	748.87 ± 25.70	69.89 ± 36.12	ND	38.50
Liver	5049.44 ± 370.61	2355.04 ± 258.05	1430.10 ± 175.39	126.35 ± 42.35	ND	33.00
Kidney	6245.27 ± 673.61	2034.47 ± 346.37	1145.28 ± 115.69	86.05 ± 41.77	ND	28.88
Lung	1884.05 ± 294.15	1681.08 ± 112.14	797.45 ± 95.81	49.18 ± 18.52	ND	30.13
Spleen	1127.80 ± 87.25	759.31 ± 47.25	271.70 ± 53.23	25.21 ± 41.14	ND	30.13
Muscle	969.02 ± 61.06	529.82 ± 54.87	ND	ND	ND	
Heart	1091.00 ± 197.10	662.17 ± 101.49	243.76 ± 46.78	ND	ND	31.50
Stomach	2290.13 ± 616.19	448.66 ± 43.56	142.33 ± 9.93	ND	ND	18.24
Large Intestine	2322.96 ± 113.41	558.10 ± 57.02	129.77 ± 23.80	ND	ND	16.90
Small Intestine	2867.77 ± 107.20	463.68 ± 120.91	57.85 ± 17.62	ND	ND	12.60
Skin/Fat	1299.60 ± 234.17	1035.96 ± 76.42	420.24 ± 36.44	25.30 ± 22.33	ND	18.24
Brain	627.02 ± 60.33	450.64 ± 41.38	ND	ND	ND	
Caecum	15540.9 ± 2038.67	563.84 ± 65.58	20.75 ± 11.12	ND	ND	7.45
Testicle	1051.96 ± 7.06	740.37 ± 77.82	331.90 ± 54.29	ND	ND	40.76
Ovary	2884.21 ± 66.88	326.55 ± 39.23	18.87 ± 6.19	ND	ND	9.63
Bone	494.69 ± 58.79	280.43 ± 56.15	ND	ND	ND	
Bladder	988.63 ± 197.38	ND	ND	ND	ND	

Note: ND, no detectable.

### 3.7 Transcriptomic data show low hepatotoxicity of CYA

#### 3.7.1 Transcriptome gene sequencing data quality control and genome sequence alignment

The results are shown in Table 4. A total of 69.31 Gb of Clean Data was obtained in this project, and the Clean Data of all samples reached more than 7.71 Gb. The percentage of Q30 bases was more than 95.66%, and the percentage of Q20 bases was more than 98.59%, and the GC content of all sample sequences was controlled to be around 50%, which indicated that the quality of data was high. Genome comparison and localisation were performed for all samples, and the reference gene source was Mus_musculus; reference genome version: GRCm39; reference genome from http://asia.ensembl.org/Mus_musculus/Info/Index. The Clean Reads of each sample were respectively compared with the specified reference genome. The matching rates ranged from 94.6% to 96.16%, indicating that the matching was very high and the data could be used for subsequent analysis.

**TABLE 4:** Transcriptome data quality statistics.

Sample	Raw reads	Clean reads	Total reads	Total mapped	Multiple mapped	Unique mapped	Error rate (%)	Q20 (%)	Q30 (%)	GC content (%)
A1	58,853,438	58,353,610	58,353,610	56,113,448 (96.16%)	4,841,804 (8.30%)	51,271,644 (87.86%)	0.0122	98.59	95.67	48.42
A2	62,056,748	61,516,420	61,516,420	59,066,051 (96.02%)	5,874,561 (9.55%)	53,191,490 (86.47%)	0.0122	98.6	95.68	48.03
A3	66,605,354	66,059,546	66,059,546	62,495,178 (94.60%)	7,652,813 (11.58%)	54,842,365 (83.02%)	0.0121	98.65	95.8	47.50
A4	64,022,618	63,524,900	63,524,900	61,035,075 (96.08%)	6,114,259 (9.62%)	54,920,816 (86.46%)	0.0121	98.68	95.89	48.00
B1	52,013,404	51,587,374	51,587,374	49,369,468 (95.70%)	5,264,250 (10.20%)	44,105,218 (85.50%)	0.0121	98.63	95.78	47.72
B2	55,354,612	54,892,216	54,892,216	52,440,677 (95.53%)	5,671,507 (10.33%)	46,769,170 (85.20%)	0.0122	98.59	95.66	47.63
B3	56,193,836	55,710,108	55,710,108	53,212,412 (95.52%)	5,552,469 (9.97%)	47,659,943 (85.55%)	0.0121	98.64	95.79	47.91
B4	51,914,814	51,480,926	51,480,926	49,385,086 (95.93%)	4,892,574 (9.50%)	44,492,512 (86.43%)	0.0122	98.6	95.68	47.80

#### 3.7.2 Total and differential gene expression analysis

**FIGURE 5:**
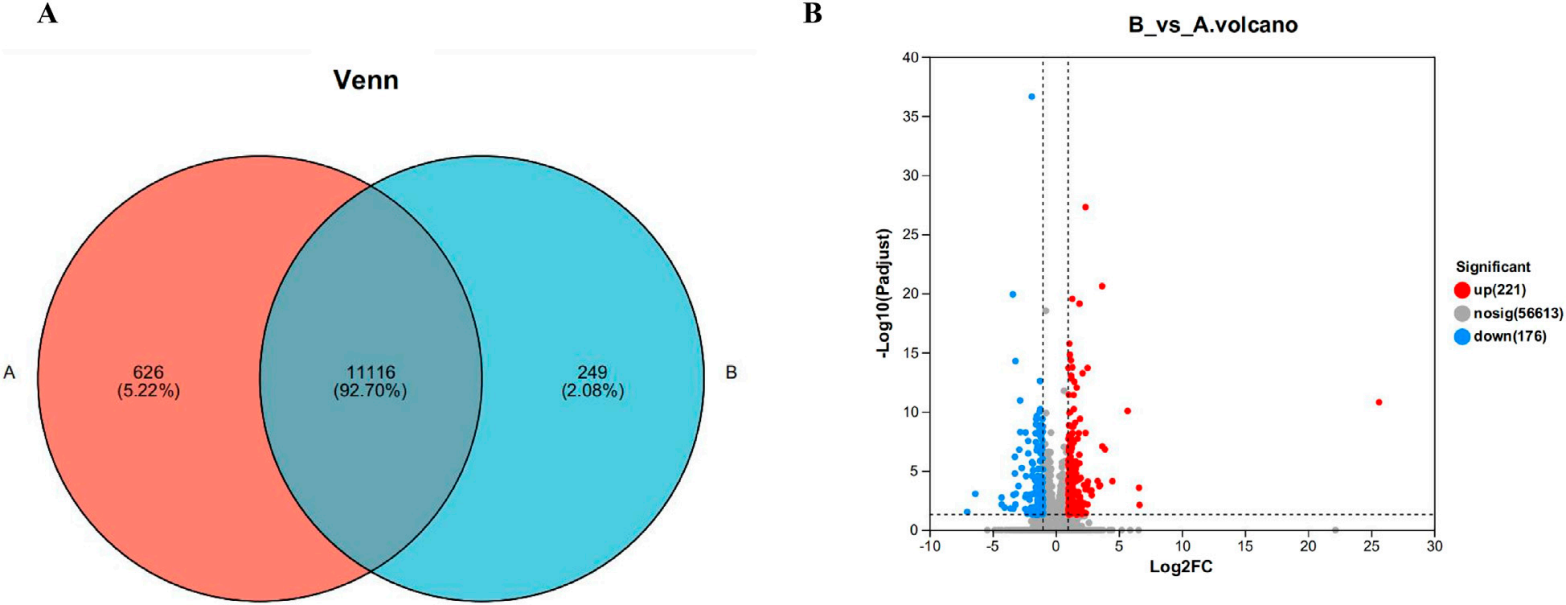
Venn diagram **(A)** and volcano map **(B)** of gene expression between liver samples of different subgroups. Where control is the A group; model is the CYA-treated group, as B group. Of these, there were 11,116 genes expressed in all groups, 626 genes expressed uniquely in the blank group, and 249 genes expressed uniquely after administration of the drug **(A)**. It can be seen that 221 genes were significantly upregulated and 176 genes were significantly downregulated in the drug group compared with the blank group **(B)**.

Statistical analysis of gene expression of samples between different subgroups was performed. The results are shown in Figure 5A. Of these, there were 11,116 genes expressed in all groups, 626 genes expressed uniquely in the blank group, and 249 genes expressed uniquely after administration of the drug.

Differently expressed genes are genes with significant differences obtained by computing the expression of all genes between different groups after transcriptome sequencing. On the Meggie Bio platform, differential gene analysis was carried out between groups by quantifying the expression results to obtain the genes that were differentially expressed between the two groups, and the differential analysis software was: DESeq2, the screening threshold was: |log2FC| ≥0 and padjust< 0.05, and the specific results are shown in Figure 5B below. It can be seen that 221genes were significantly upregulated and 176 genes were significantly downregulated in the drug group compared with the blank group, further indicating only a very small number of genes showed significant changes in expression after drug administration.

#### 3.7.3 Analysis of the functional annotation of the differential genes after CYA administration

**FIGURE 6:**
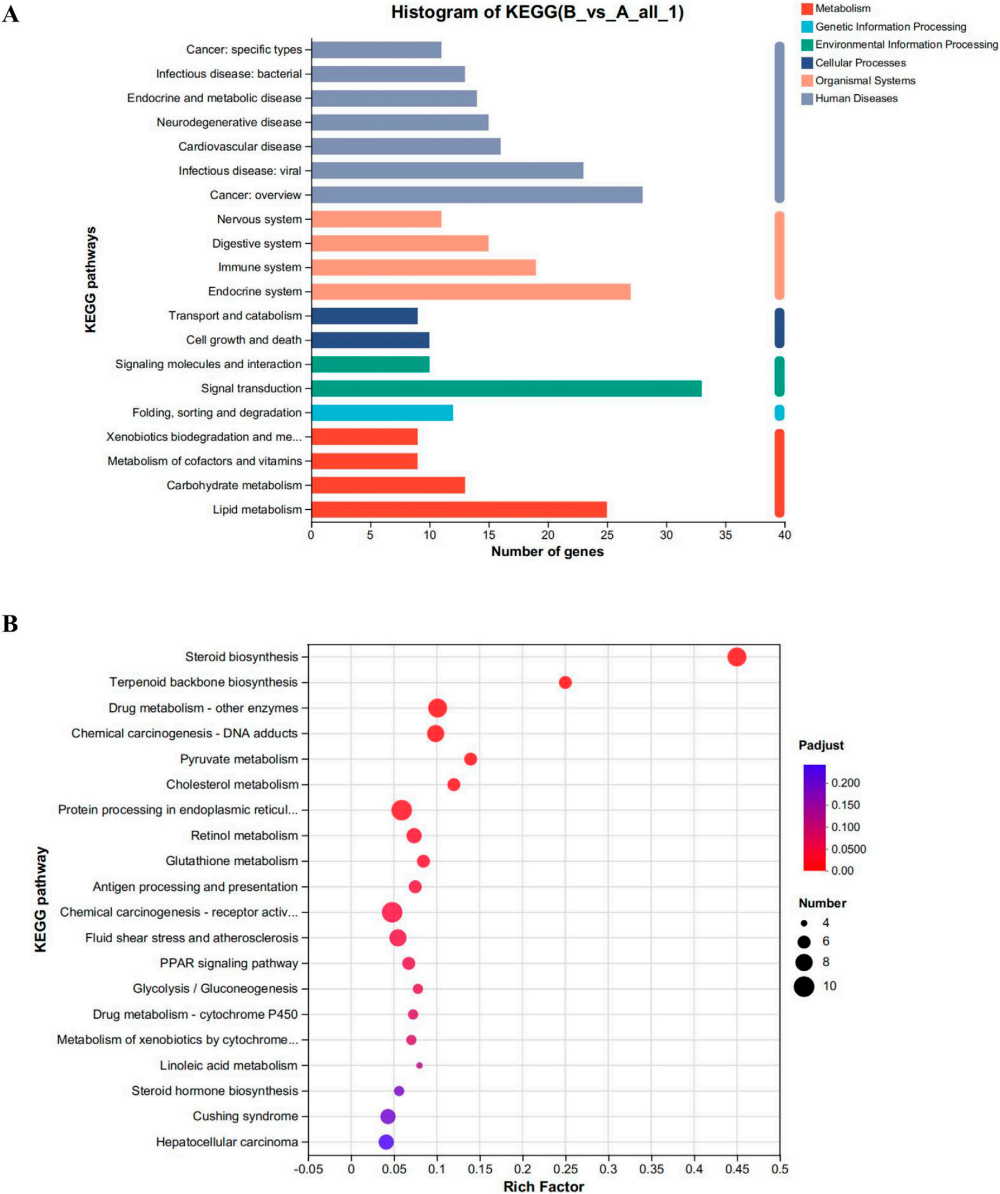
**(A)** Functional annotation analysis plot and **(B)** Kegg functional enrichment analysis plot of differential genes in the CYA-treated group vs. control group. It can be seen that administration of CYA had a significant effect on lipid metabolism **(A)** and may influence steroid biosynthesis and metabolism **(B)**.

To examine the impact of CYA on the liver, we employed the Meggie Bio platform to establish differential gene expression sets between the CYA administration group and the control group (drug vs. control). Subsequently, we subjected the target gene sets obtained from the differential gene sets to target gene function annotation analysis and target gene function enrichment analysis, utilizing the target gene set analysis function. Figure 6 presents the results of these analyses.

The analysis of the functions of the differential gene set, as shown in Figure 6, reveals several interesting findings. Analysis of Figure 6A reveals that the administration of CYA had a significant impact on lipid metabolism. The Endocrine system exhibited the highest number of differential genes among the various systems. Moreover, KEGG enrichment analysis highlighted Steroid biosynthesis as the pathway most affected by the drug (Figure 6B). This finding was unexpected and suggests that the administration of CYA may influence steroid synthesis and metabolism, which has been rarely reported in previous literature. Furthermore, in order to assess the toxicity of CYA, it is necessary to analyze differential genes associated with Cell growth and death in Cellular Processes. This analysis will help determine if the administration of CYA affects the expression of apoptosis-related genes in hepatocytes. Therefore, it is crucial to further investigate differential genes related to cell processes and lipid metabolism.

#### 3.7.4 Analysis of Kegg functional enrichment and expression of cell process and lipid metabolism related differential genes

**FIGURE 7:**
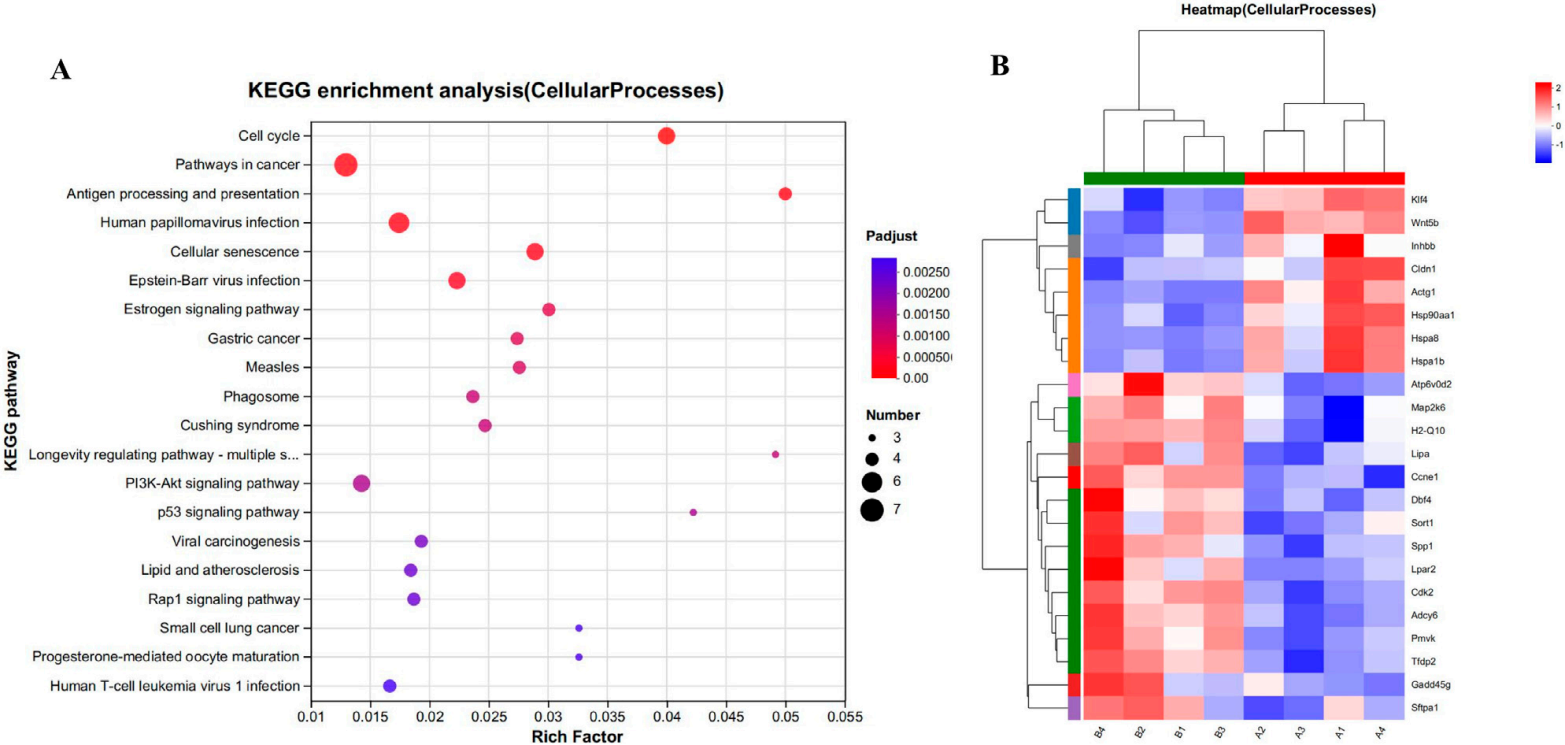
**(A)** Kegg functional enrichment analysis plot of differentially regulated genes in cellular processes. **(B)** The hierarchical clustering heat maps of cellular processes-related genes in model vs. drug groups. Where A (A1-A4) is control group; B (B1-B4) is CYA-treated group.

To explore the potential impact of CYA administration on apoptosis in liver cells, we performed KEGG functional enrichment analysis on the set of differential genes associated with cell processes in both the control group (Group A; A1, A2, A3, A4) and the CYA-treated group (Group B; B1, B2, B3, B4). The results of this analysis are displayed in Figure 7. Among the 23 significantly different genes, none were found to be associated with the process of apoptosis. This result indicates that the administration of CYA is unlikely to cause hepatotoxicity, as there is no evidence of genes related to apoptosis being affected.

**FIGURE 8:**
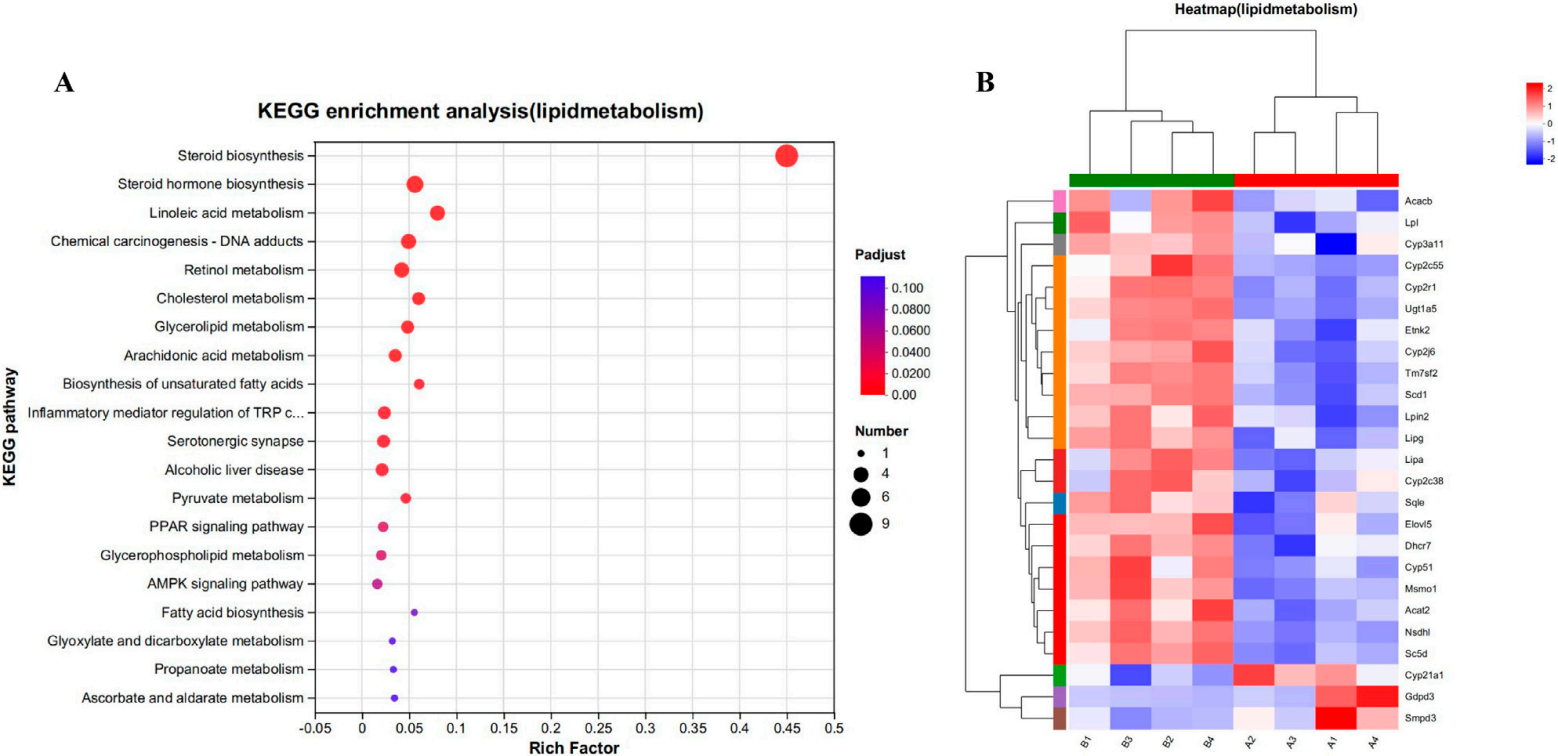
**(A)** Kegg functional enrichment analysis plot of differentially regulated genes in lipid metabolism. **(B)** The hierarchical clustering heat maps of lipid metabolism-related genes in model vs. drug groups. Where A (A1-A4) is control group; B (B1-B4) is CYA-treated group.

Unexpectedly, the enrichment analysis of differential genes following CYA administration revealed a significant association with lipid metabolism. After CYA administration, a total of 25 genes associated with lipid metabolism-related pathways exhibited significant changes (Figure 8). These genes include cytochrome P450 family-related genes (CYP51, CYP21A1, CYP2C55, CYP2R1, CYP2C38, CYP2J6, CYP3A11) and genes related to steroid biosynthesis and the steroid hormone pathway (SQLE, CYP51, TM7SF2, MSMO1, NSDHL, SC5DL, CYP2R1, DHCR7, LIPA, CYP21A1, CYP2C55, CYP2C38, CYP3A11, UGT1A5). Notably, these genes were predominantly downregulated after CYA administration, suggesting that the drug may impact the synthesis of steroid hormones in organisms.

## 4 Discussion

**FIGURE 9:**
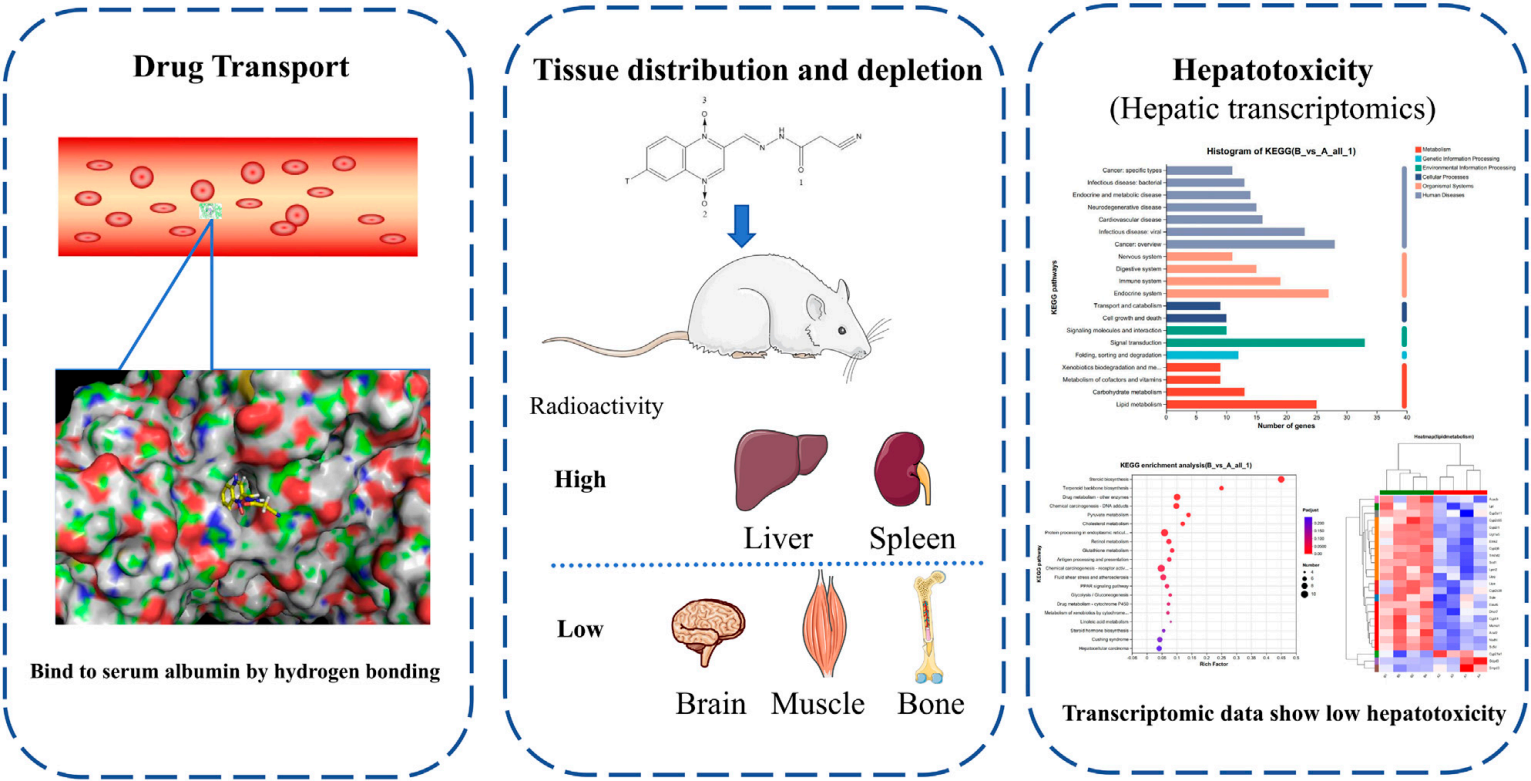
In this study, we investigate the transport of CYA in the bloodstream, its distribution in various tissues and organs, and the impact of the drug on the liver, which is a key organ involved in the enrichment and distribution of the drug. The research findings suggest that CYA is a low toxicity and effective synthetic antibacterial agent.

In this article, we investigate the transport of CYA in the bloodstream, its distribution in various tissues and organs, and the impact of the drug on the liver, which is a key organ involved in the enrichment and distribution of the drug (Figure 9). The research findings suggest that CYA is a low toxicity and effective synthetic antibacterial agent.

The binding of blood plasma proteins to drug affects drug distribution. The interaction between CYA and BSA was evaluated using fluorescence spectroscopy. Our data clearly indicated that the fluorescence intensity of BSA decreased in the presence of CYA and that CYA interacted with BSA. Though the Stern–Volmer equation, we concluded that the fluorescence quenching of BSA was initiated by BSA–CYA complex formation rather than dynamic collision (Rychen et al., 2017). The number of binding sites was only one. The BSA–CYA complex was unstable and became partly decomposed when the temperature was increased. In addition, the synchronous fluorescence spectra showed that the tryptophan residues but not the tyrosine residues participated in the interaction between cyadox and BSA. CD was used to determine the secondary structure of BSA binding with CYA. An obvious secondary structural transformation of BSA was observed (Figure 4), indicating a strong affinity for CYA (Chen et al., 2022). The secondary protein conformation of BSA changed with an α-helix increase and a β-strand decrease after CYA binding to BSA (Sharma et al., 2020). Computational molecular docking was employed to improve the understanding of the interaction between BSA and CYA. The present study showed that CYA can bind within the pocket of BSA (Figure 5). Specifically, hydrogen bonds formed between O (2) of CYA and ARG208, O (3) of CYA and LEU480, VAL481 in the pocket. The complexation of BSA–CYA was stabilized primarily by hydrogen bond interactions. The results indicate that CYA is primarily transported in the bloodstream through serum albumin, which subsequently distributes the drug to various organs and tissues throughout the body of the animal.

The use of the radioactive tracer method provides a distinct approach for quantifying the overall presence of drug-related molecules. This methodology allowed us to effectively observe the excretion and distribution patterns of CYA drugs within the tissues. The present study described the disposition of CYA in rats orally administered with [^3^H]-CYA at different sequences. The radioactivity was mainly excreted in the feces (60.25%) and then in the urine (34.81%). In addition, 91.28% of the administration amount of the drug-related radioactivity was excreted during the first 24 h after administration, and that excreted at 0–12 and 12–24 h post-dose accounted for 21.03% and 70.25% of the total dose, respectively, which suggested the rapid and complete excretion of CYA in rats. In the present study, we administered [^3^H]-CYA orally to six groups of rats for seven consecutive days. Following administration, we observed a widespread distribution of radioactivity throughout the body, with maximum concentrations reached at 6 h in all analyzed organs and tissues. However, by day 14 after the dose administration, radioactivity became undetectable in the blood, organs, and tissues, suggesting rapid elimination of the drug in the rats. Furthermore, the radioactivity in all analyzed organs and tissues declined rapidly after a 6-h withdrawal period. Notably, the alimentary canal tissues, including the cecum, large intestine, small intestine, and stomach, exhibited relatively high levels of total radioactivity at earlier time points, but with a short residual time. Conversely, the liver showed the highest radioactivity levels from 1 to 7 days, indicating that the drug may persist in the liver for an extended period. Due to the potential risk of CYA persisting in the liver for an extended period and being enriched at high concentrations, which could potentially result in hepatotoxicity, we proceeded with the subsequent phase of our study.

To comprehensively understand the impact of CYA administration on the liver, we employed transcriptomics to analyze the genes associated with significant changes in the liver of mice following drug administration. This approach allowed us to gain detailed insights into the effects of CYA on liver function at the genetic level. Firstly, we conducted an analysis of genes associated with cellular processes and observed that only 23 genes exhibited significant changes after CYA administration (Figure 7). This finding suggests that the administration of CYA has minimal impact on the vital activities of liver cells. Additionally, upon further analysis of these 23 significantly altered genes, we found no genes related to apoptosis. This result provides evidence that CYA demonstrates low hepatotoxicity. Interestingly, on the other hand, our functional enrichment analysis of the genes with significant changes revealed their enrichment in pathways associated with lipid metabolism (Figure 6), which are closely linked to the steroid synthesis pathway. This suggests that CYA may have unexpected roles and potentially influences lipid metabolism in addition to its intended effects. CYA is a Quinoxaline 1,4-dioxide derivatives. As a Quinoxaline 1,4-dioxide derivative, we conducted a thorough literature search to investigate any existing literature on the relationship between Quinoxaline derivatives and steroid synthesis. A study revealed that the mRNA expression levels of StAR, P450scc, and 17β-HSD in the testis were significantly reduced in the treatment group exposed to 275 mg/kg of Mequindox (MEQ), compared to the control group. However, in the group exposed to 110 mg/kg of MEQ, the mRNA expression levels of AR and 3β-HSD were significantly increased compared to the control group (Ihsan et al., 2011). These findings suggest that MEQ, a Quinoxaline 1,4-dioxide derivative, can affect the mRNA expression of these genes involved in steroid synthesis in the testis. These effects on gene expression are associated with testicular toxicity. Despite the transcriptomic data showing no significant decrease in the expression of reported genes (StAR, P450scc, and 17β-HSD) following CYA administration, the potential reproductive toxicity of CYA cannot be disregarded due to the downregulation of genes associated with steroid biosynthesis. After CYA administration, several genes involved in steroid biosynthesis exhibited significant changes. CYP51 is associated with antifungal activity (Zhang et al., 2023), while CYP51, TM7SF2, SQLE, and MSMO1 are involved in cholesterol biosynthesis (Zhang et al., 2019; Allimuthu et al., 2019; Jia et al., 2024). NSDHL, SC5DL, and CYP3A11 are associated with immunity (Xie et al., 2023; Smith et al., 2022; Ashino et al., 2023). Additionally, CYP2R1 and DHCR7 are related to vitamin D metabolism (Harishankar et al., 2021; Sun et al., 2023), LIPA is associated with non-alcoholic fatty liver disease (Fang et al., 2024), and CYP21A1 affects animal development (You et al., 2024). While there have been no reports of significant changes in these genes being associated with reproductive toxicity, it is important to further investigate the potential reproductive toxicity of CYA.

## 5 Conclusion

In summary, this study investigated the interaction of bovine serum albumin (BSA) with CYA, mass balance, distribution, and depletion of CYA in the rats following oral administration of [^3^H]-CYA at different sequences. Our data suggest that the fluorescence quenching of BSA is initiated by BSA–CYA complex formation rather than dynamic collision. Hydrogen bonds formed between O (2) of CYA and ARG208, O (3) of CYA and LEU480, VAL481. The secondary protein conformation of BSA changed with an α-helix increase and a β-strand decrease after CYA binding to BSA. Results showed a rapid excretion, widespread distribution, and rapid depletion in the rats. Furthermore, CYA was found to accumulate at high levels in the liver and exhibit a long half-life. The analysis of mouse liver transcriptome revealed that CYA administration resulted in low hepatotoxicity but significant alterations in genes related to sterol synthesis. These findings suggest that CYA has an impact on lipid metabolism. The current study could serve as a basis for conducting further studies on the use of CYA in food animals, understanding the pharmacologic and toxicologic effects of CYA on food animals, and evaluating the food safety of CYA.

## Data Availability

The datasets presented in this study can be found in online repositories. The names of the repository/repositories and accession number(s) can be found below: https://www.ncbi.nlm.nih.gov/, BioProject: Number: PRJNA1082257.

## References

[B1] Allimuthu D. Hubler Z. Najm F. J. Tang H. Bederman I. Seibel W. (2019). Diverse chemical scaffolds enhance oligodendrocyte formation by inhibiting CYP51, TM7SF2, or EBP. Cell Chem. Biol. 26, 593–599. 10.1016/j.chembiol.2019.01.004 30773481 PMC6474808

[B2] Ashino T. Nakamura Y. Ohtaki H. Iwakura Y. Numazawa S. (2023). Downregulation of the gene expression of Cyp2c29 and Cyp3a11 by cecal ligation and puncture-induced sepsis is associated with interleukin-6. Int. Immunopharmacol. 117, 110039. 10.1016/j.intimp.2023.110039 36944277

[B3] Ashraf G. M. Gupta D. D. Alam M. Z. Baeesa S. S. Alghamdi B. S. Anwar F. (2022). Unravelling binding of human serum albumin with galantamine: spectroscopic, calorimetric, and computational approaches. ACS Omega 7, 34370–34377. 10.1021/acsomega.2c04004 36188253 PMC9521020

[B4] Chen R. He R.-J. Guo D. Zhang Z.-F. Zhang W.-G. Fan J. (2022). Interactions of diclazuril enantiomers with serum albumins: multi-spectroscopic and molecular docking approaches. J. Mol. Recognit. 35, e2948. 10.1002/jmr.2948 35094438

[B5] Fang C. Liu S. Yang W. Zheng G. Zhou F. Gao X. (2024). Exercise ameliorates lipid droplet metabolism disorder by the PLIN2–LIPA axis-mediated lipophagy in mouse model of non-alcoholic fatty liver disease. Biochimica Biophysica Acta (BBA) - Mol. Basis Dis. 1870, 167045. 10.1016/j.bbadis.2024.167045 38306800

[B6] Harishankar M. Sampath P. Sriram M. Raghuraman R. Athikesavan V. Chinnayan P. (2021). Association of CYP2R1 gene polymorphisms in pulmonary tuberculosis. Meta Gene 28, 100875. 10.1016/j.mgene.2021.100875

[B7] Huang L. Wang Y. Tao Y. Chen D. Yuan Z. (2008). Development of high performance liquid chromatographic methods for the determination of cyadox and its metabolites in plasma and tissues of chicken. J. Chromatogr. B-ANALYTICAL Technol. Biomed. LIFE Sci. 874, 7–14. 10.1016/j.jchromb.2008.08.013 18838344

[B8] Huang L. L. Qiu Y. M. Sun L. L. Li J. Pan Y. H. Wang Y. L. (2018). Dietary exposure assessment of cyadox based on tissue depletion of cyadox and its major metabolites in pigs, chickens, and carp. J. VETERINARY Pharmacol. Ther. 41, 125–136. 10.1111/jvp.12440 29194660

[B9] Huang Q. Ihsan A. Guo P. Luo X. Cheng G. Hao H. (2016). Evaluation of the safety of primary metabolites of cyadox: acute and sub-chronic toxicology studies and genotoxicity assessment. Regul. Toxicol. Pharmacol. 74, 123–136. 10.1016/j.yrtph.2015.11.011 26617409

[B10] Huang Z.-Y. Li X.-Y. Hu L.-Y. Bai A.-M. Hu Y.-J. (2022). Comparative study of two antipsychotic drugs binding to human serum albumin: by multispectroscopic and molecular docking methods. J. Mol. Liq. 365, 120084. 10.1016/j.molliq.2022.120084

[B11] Ihsan A. Wang X. Huang X.-j. Liu Y. Liu Q. Zhou W. (2010). Acute and subchronic toxicological evaluation of Mequindox in Wistar rats. Regul. Toxicol. Pharmacol. 57, 307–314. 10.1016/j.yrtph.2010.03.011 20371258

[B12] Ihsan A. Wang X. Liu Z. Wang Y. Huang X. Liu Y. (2011). Long-term mequindox treatment induced endocrine and reproductive toxicity via oxidative stress in male Wistar rats. Toxicol. Appl. Pharmacol. 252, 281–288. 10.1016/j.taap.2011.02.020 21377486

[B13] Ihsan A. Wang X. Tu H.-G. Zhang W. Dai M.-H. Peng D.-P. (2013). Genotoxicity evaluation of Mequindox in different short-term tests. FOOD Chem. Toxicol. 51, 330–336. 10.1016/j.fct.2012.10.003 23063596

[B14] Jia Q. Cao Y. Zhang M. Xing Y. Xia T. Guo Y. (2024). miR-19b-3p regulated by estrogen controls lipid synthesis through targeting MSMO1 and ELOVL5 in LMH cells. Poult. Sci. 103, 103200. 10.1016/j.psj.2023.103200 37939591 PMC10665931

[B15] Laulloo S. J. Caumul P. Joondan N. Jawaheer S. Parboteeah S. Dyall S. D. (2022). A study of the antibacterial activities and the mode of action of L-methionine and L-cystine based surfactants and their interaction with bovine serum albumin using fluorescence spectroscopy and *in silico* modelling. BIOINTERFACE Res. Appl. Chem. 12, 7356–7375. 10.33263/BRIAC126.73567375

[B16] Li Y. F. Zhao N. Zeng Z. K. Gu X. Y. Fang B. H. Yang F. (2013). Tissue deposition and residue depletion of cyadox and its three major metabolites in pigs after oral administration. J. Agric. FOOD Chem. 61, 9510–9515. 10.1021/jf4028602 24050441

[B17] Liu Q. Lei Z. Cui L. Zhang J. Awais I. Dai M. (2017). A two-year dietary carcinogenicity study of cyadox in Sprague-Dawley rats. Regul. Toxicol. Pharmacol. 87, 9–22. 10.1016/j.yrtph.2017.04.011 28454720

[B18] Liu Z. Y. Huang L. L. Dai M. H. Chen D. M. Tao Y. F. Wang Y. L. (2009). Metabolism of cyadox in rat, chicken and pig liver microsomes and identification of metabolites by accurate mass measurements using electrospray ionization hybrid ion trap/time-of-flight mass spectrometry. RAPID Commun. MASS Spectrom. 23, 2026–2034. 10.1002/rcm.4106 19504544

[B19] Lu H. A. Li Z. S. Zhou Y. S. Jiang H. Liu Y. F. Hao C. C. (2022). Horizontal comparison of “red or blue shift” and binding energy of six fluoroquinolones: fluorescence quenching mechanism, theoretical calculation and molecular modeling method. SPECTROCHIMICA ACTA PART A-MOLECULAR Biomol. Spectrosc. 278, 121383. 10.1016/j.saa.2022.121383 35597157

[B20] Marathe P. H. Shyu W. C. Humphreys W. G. (2004). The use of radiolabeled compounds for ADME studies in discovery and exploratory development. Curr. Pharm. Des. 10, 2991–3008. 10.2174/1381612043383494 15379664

[B21] Rychen G. Aquilina G. Azimonti G. Bampidis V. Bastos M. L. Bories G. (2017). Guidance on the assessment of the safety of feed additives for the consumer. EFSA J. 15, e05022. 10.2903/j.efsa.2017.5022 32625312 PMC7009902

[B22] Sharma K. Yadav P. Sharma B. Pandey M. Awasthi S. K. (2020). Interaction of coumarin triazole analogs to serum albumins: spectroscopic analysis and molecular docking studies. J. Mol. Recognit. 33, e2834. 10.1002/jmr.2834 32017307

[B23] Smith T. Rohaim M. A. Munir M. (2022). Mapping molecular gene signatures mediated by SARS-COV-2 and large-scale and genome-wide transcriptomics comparative analysis among respiratory viruses of medical importance. Mol. Cell. Probes 64, 101820. 10.1016/j.mcp.2022.101820 35504488 PMC9054707

[B24] Sun L. Lu J. Yao D. Li X. Cao Y. Gao J. (2023). Effect of DHCR7 for the co-occurrence of hypercholesterolemia and vitamin D deficiency in type 2 diabetes: perspective of health prevention. Prev. Med. 173, 107576. 10.1016/j.ypmed.2023.107576 37329988

[B25] Wang X. Zhou W. Ihsan A. Chen D. Cheng G. Hao H. (2015). Assessment of thirteen-week subchronic oral toxicity of cyadox in Beagle dogs. Regul. Toxicol. Pharmacol. 73, 652–659. 10.1016/j.yrtph.2015.09.023 26408151

[B26] Wu H. X. Li L. X. Shen J. Z. Wang Y. Liu K. L. Zhang S. X. (2012). *In vitro* metabolism of cyadox in rat, chicken and swine using ultra-performance liquid chromatography quadrupole time-of-flight mass spectrometry. J. Pharm. Biomed. ANALYSIS 67-68, 175–185. 10.1016/j.jpba.2012.04.004 22565170

[B27] Xie L. Liu M. Cai M. Huang W. Guo Y. Liang L. (2023). Regorafenib enhances anti-tumor efficacy of immune checkpoint inhibitor by regulating IFN-γ/NSDHL/SREBP1/TGF-β1 axis in hepatocellular carcinoma. Biomed. and Pharmacother. 159, 114254. 10.1016/j.biopha.2023.114254 36669362

[B28] Xu N. Huang L. L. Liu Z. L. Pan Y. H. Wang X. Tao Y. F. (2011). Metabolism of cyadox by the intestinal mucosa microsomes and gut flora of swine, and identification of metabolites by high-performance liquid chromatography combined with ion trap/time-of-flight mass spectrometry. RAPID Commun. MASS Spectrom. 25, 2333–2344. 10.1002/rcm.5119 21766376

[B29] You Z. Yuan J. Wang Y. Sun Y. Ni A. Li Y. (2024). Integrated transcriptomic analysis on chicken ovary reveals CYP21A1 affects follicle granulosa cell development and steroid hormone synthesis. Poult. Sci. 103, 103589. 10.1016/j.psj.2024.103589 38471223 PMC11067781

[B30] Zhang C. Zhang H. Zhang M. Lin C. Wang H. Yao J. (2019). OSBPL2 deficiency upregulate SQLE expression increasing intracellular cholesterol and cholesteryl ester by AMPK/SP1 and SREBF2 signalling pathway. Exp. Cell Res. 383, 111512. 10.1016/j.yexcr.2019.111512 31356817

[B31] Zhang R. Wang Y. Wu A. Wang J. Zhang J. (2023). Strategies of targeting CYP51 for IFIs therapy: emerging prospects, opportunities and challenges. Eur. J. Med. Chem. 259, 115658. 10.1016/j.ejmech.2023.115658 37480712

[B32] Zheng M. Jiang J. Wang J. P. Tang X. Q. Ouyang M. Deng Y. Q. (2011). The mechanism of enzymatic and non-enzymatic N-oxide reductive metabolism of cyadox in pig liver. XENOBIOTICA 41, 964–971. 10.3109/00498254.2011.593207 21745143

[B33] Zhou B. J. Zhou H. Xu L. L. Cai R. R. Chen C. L. Chi B. Z. (2022). An insight into the interaction between Indisulam and human serum albumin: spectroscopic method, computer simulation and *in vitro* cytotoxicity assay. Bioorg. Chem. 127, 106017. 10.1016/j.bioorg.2022.106017 35841666

